# Non-binary Colour Modulation for Display Device Based on Phase Change Materials

**DOI:** 10.1038/srep39206

**Published:** 2016-12-19

**Authors:** Hong-Kai Ji, Hao Tong, Hang Qian, Ya-Juan Hui, Nian Liu, Peng Yan, Xiang-Shui Miao

**Affiliations:** 1 School of Optical and Electronic Information, Huazhong University of Science and Technology, Wuhan 430074, China; 2 Wuhan National Laboratory for Optoelectronics, Huazhong University of Science and Technology, Wuhan 430074, China

## Abstract

A reflective-type display device based on phase change materials is attractive because of its ultrafast response time and high resolution compared with a conventional display device. This paper proposes and demonstrates a unique display device in which multicolour changing can be achieved on a single device by the selective crystallization of double layer phase change materials. The optical contrast is optimized by the availability of a variety of film thicknesses of two phase change layers. The device exhibits a low sensitivity to the angle of incidence, which is important for display and colour consistency. The non-binary colour rendering on a single device is demonstrated for the first time using optical excitation. The device shows the potential for ultrafast display applications.

In the modern world, it is becoming increasingly important to develop electronic displays for devices ranging from mobile phones to wearable devices. Currently, there is a strong drive towards the development of ultrathin displays with high resolution and fast response time and with good viewability in bright sunlight in which backlight or emissive devices perform very poorly. Recently, a reflective-type display device that relies on the strong Fabry–Perot-type interference and is based on phase change materials (Ge_2_Sb_2_Te_5_) has been demonstrated^1^,^2^. In this device, colours can be obtained using nanometric scales and low dimensionality^1^. The display device could provide bistability and ultrafast response times because Ge_2_Sb_2_Te_5_ (GST) can switch rapidly between the amorphous and the crystalline states — both of which are stable at room temperature^3^,^4^ — in response to an external excitation such as heat, light or electrical current^5^,^6^. Furthermore, the device is extremely scalable^7^,^8^, operates with low power consumption and can be easily manipulated both on rigid and flexible surfaces^1^.

Despite its tremendous advantages, the key drawback of the display device using GST-based thin films relates to its colour depth modulation capability. Only two colours can be tuned via the full crystallization of the few-nanometres-thick GST layer without the contribution provided by changing the thickness of indium tin oxide (ITO) beneath the GST. Previous studies tend to use single phase change material (PCM) as a highly absorbing dielectric layer to realize colour depth modulation, for example, Ag_3_In_4_Sb_76_Te_17_ (AIST)^9^ and two ultrathin GST films^10^. However, the method based on two ultrathin GST films needs a barrier layer to avoid interdiffusion, which will adversely interfere with the amorphization during actual device operation^10^ and is unstable because it need accurate thickness control. In this work, we demonstrate that a better colour modulation, i.e., multiple colours, can be achieved by exploiting different stacked chalcogenide layers (Sb_2_Te_3_ and GeTe) that are sandwiched between two ITO layers and the dependency of degree crystallization on applied laser power. The sequential crystallization of different phase change films can be attained without a barrier layer, resulting in multicolour changing. Differences in the physical properties of these two PCMs contribute to the multiple colour appearances. We present the first demonstration of the structure having a low sensitivity to the angle of incidence, which is very important for displays. We further demonstrate that the colour is clearly and continuously modulated on a single device by laser illumination without the need for colour filters or a spatially modulated colour scheme. Finally, we present that nearly the entire colour gamut can be attained by combining with different thicknesses of the bottom ITO layer and selective crystallization of PCM layers.

## Results

### Device schematic

**Figure 1:**
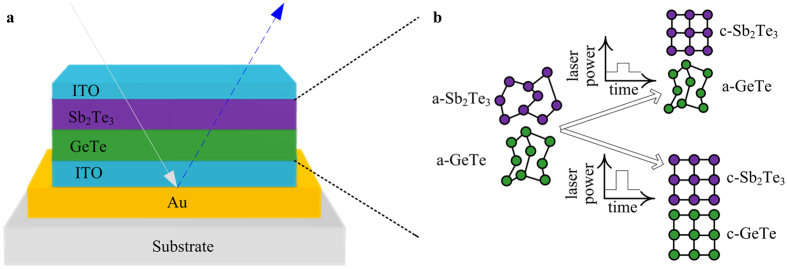
High contrast multicolour display device using double layer PCMs. (**a**) Schematic of thin film materials comprising ITO/Sb_2_Te_3_/GeTe/ITO/Au. (**b**) Schematic of operation mechanism for sequential crystallization of double layer PCMs.

Figure 1a shows the schematic of the tunable multicolour device. Multi-layered films were deposited on an Si substrate covered with a 1μm thick layer of thermally grown SiO_2_. Double layer PCMs (Sb_2_Te_3_ and GeTe) are sandwiched between two ITO layers and are deposited on a reflective Au layer (30 nm ITO/2 nm Sb_2_Te_3_/6 nm GeTe/35 nm ITO/100 nm Au). ITO was selected to as the electrode material because of its extreme stability at high temperatures^11^, high transmission in the visible spectrum and remarkable electrical conductance^12^. The thicknesses of PCMs are optimized to maximize the optical contrast (see below). The upper ITO layer prevents the evaporation and oxidation of the PCMs and acts as the top electrode in the display device driven by electrical current^1^. The bottom reflective surface Au film is sufficiently thick for preventing the penetration of light to the substrate.

Figure 1b provides an illustration of the sequential crystallization of double layer PCMs. The green and purple atoms represent the GeTe and Sb_2_Te_3_ layer, respectively (a denotes amorphous, c denotes crystalline). Sputtered Sb_2_Te_3_ and GeTe films are amorphous^13^,^14^. The crystallization temperature (Tc) of Sb_2_Te_3_ is much lower than that of GeTe^15^,^16^,^17^; thus, when a external stimulus (i.e., heat, laser or current) is applied, the priority of Sb_2_Te_3_ crystallization is guaranteed when the temperature reaches the Tc of Sb_2_Te_3_ but is lower than that for GeTe^18^,^19^. Owing to the big Tc difference between Sb_2_Te_3_ and GeTe, it is easier and more stable to realize multistate phase combinations and continuous colour modulation (see below) and also improve the optical or electrical operation window compared with the device based on two ultrathin GST films with different thicknesses. The barrier layer in Yoo and colleagues’ device is the key component to obtain multiple colours. However, during the reset (amorphization) process, its thermal insulation effect will adversely interfere with the amorphization during actual device operation, resulting in not being able to switch repeatedly^10^. In our device selective phase transition of each PCM layer can be attained without a thermal/diffusion barrier layer because at the interface between Sb_2_Te_3_ and GeTe, Ge–Te and Sb–Te bonds can be formed which provided a barrier to slow down the exchange and the interdiffusion^20^. A change in phase causes the change in the reflective index of the two PCM layers and modulates the reflective spectrum of the entire stack. As a result, a colour change in the entire film is induced by the sequential crystallization of double layer PCMs when incident light is reflected back.

### Simulated method of reflectivity

To quantify the colour change, the transfer matrix method^21^ is used to calculate the reflectivity. The reflectivity is


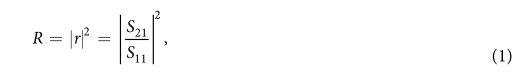


where S_21_ and S_11_ are transfer matrix elements,


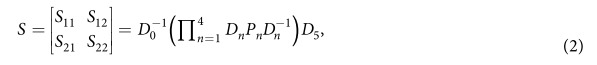


with,


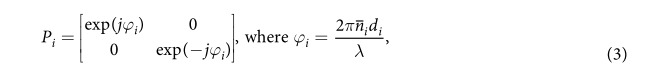


for transverse-electric (TE) polarization,


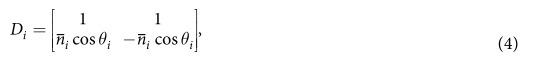


for transverse-magnetic (TM) polarization,


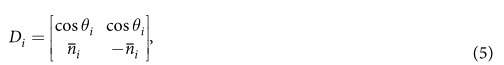


where i = 0, 1, 2, 3, 4, 5 for air, top ITO, Sb_2_Te_3_, GeTe, bottom ITO and Au, respectively. In equation (3), (4) and (5), 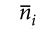 is the complex refractive index of medium i, and d_i_ is the thickness of medium i. In equation (4) and (5), *θ*_*i*_ is the incident angle in the medium i.

### Device properties

**Figure 2:**
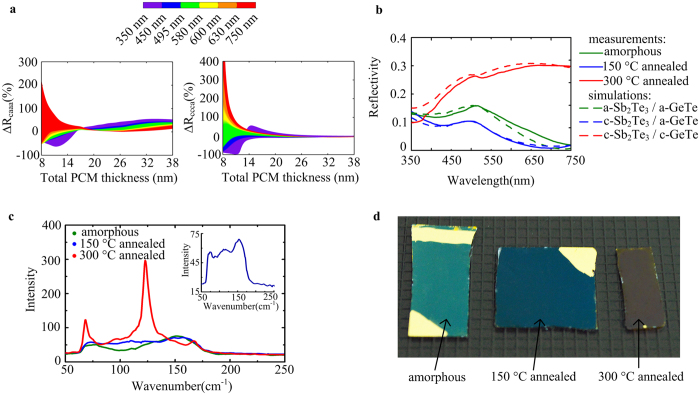
(**a**) Optical contrast between different phases of PCMs is simulated at a visible wavelength with increasing thickness of Sb_2_Te_3_ and GeTe layers. (**b**) The measured reflectivity spectra of samples shown in d correspond to the simulated reflectivity spectra. (**c**) Sequential crystallization of Sb_2_Te_3_ and GeTe is confirmed using Raman spectroscopy measurements. (**d**) Photograph of as-deposited amorphous, 150 °C annealed and 300 °C annealed samples demonstrates the high contrast colour change via sequential crystallization of Sb_2_Te_3_ and GeTe. No postproduction colour is added to enhance the contrast.

The overall optical properties of the stack are greatly influenced by the thicknesses of the PCM^1^. Hence, to maximize the optical contrast, we calculate the reflectivity change between the different phases of PCMs (see Fig. 2a) while gradually increasing the thickness of Sb_2_Te_3_ (from 2 nm to 17 nm) and GeTe (from 6 nm to 21 nm). The total PCM thickness in the stack increases from 8 nm to 38 nm. In the case of the samples shown in Fig. 2d, the thicknesses of Sb_2_Te_3_ and GeTe are 2 nm and 6 nm, respectively (these are the minimum thicknesses that we could sputter reliably using our facilities). The percentage change in reflectivity at the visible wavelength is plotted as ∆R_caaa_(%) = (R_ca_ − R_aa_)/R_aa_ × 100 and ∆R_ccca_(%) = (R_cc_ − R_ca_)/R_ca_ × 100, where R_aa_ represents the reflectivity when the Sb_2_Te_3_ and GeTe layers are both amorphous, R_ca_ is the reflectivity when the Sb_2_Te_3_ layer is crystalline and the GeTe layer is amorphous, and R_cc_ is the reflectivity when the Sb_2_Te_3_ and GeTe layers are both crystalline. Figure 2a shows that the red components are maximum for ∆R_caaa_ and ∆R_ccca_ with the remaining wavelengths weakly increasing when the total thickness of the Sb_2_Te_3_ and GeTe layers is 8 nm. With the increasing thickness of Sb_2_Te_3_ and GeTe, the optical reflectivity change greatly decreased. As a result, an ultrathin PCM enhances the optical contrast significantly.

To evaluate the feasibility of multistate colour changing, the sample was first annealed at 150 °C for 10 min in a furnace to fully crystallize the Sb_2_Te_3_ and ensure that the GeTe is still amorphous as shown in Fig. 1b. Then, the fully crystalline (meaning that Sb_2_Te_3_ and GeTe are both crystalline) sample was obtained after annealing at 300 °C for 10 min in a furnace. The appreciable colour change of these samples when the double layer PCMs crystallize sequentially is also shown in Fig. 2d. The presence of tunable three colours shows the potential for the use of these materials as individual pixels in a display device. Each pixel can display three colours without the need for colour filters or a spatially modulated colour scheme.

Then, the reflectivity spectra of samples were measured using a spectrometer (Lambda 750 S, Perkin Elmer, USA). As shown in Fig. 2b, excellent agreement between the measured and simulated reflectivity spectra was obtained for the samples shown in Fig. 2d. To prove that the colour change is associated with the phase change of Sb_2_Te_3_ and GeTe, i.e., sequential crystallization, Raman spectroscopy measurements (LabRAM HR800, Horiba Jobin Yvon, France) were performed on the three samples (Fig. 2c). The Raman peaks of the 150 °C annealed sample (shown clearly in the inset of Fig. 2c) are observed at 74 cm^−1^, 116 cm^−1^ and 155 cm^−1^, which are in good agreement with the peaks corresponding to crystalline Sb_2_Te_3_^22^,^23^,^24^. However, the Raman peak positions of the 300 °C annealed sample are slightly different from those of the purely crystalline GeTe^25^,^26^. Because the crystallization temperature and time increase for the ultrathin GeTe^27^,^28^, the majority of the GeTe layer is crystalline while a small fraction remains amorphous. At the same time, the Raman spectrum of the 300 °C annealed sample contains the peaks of not only the GeTe but also the crystalline Sb_2_Te_3_. A photograph of the as-deposited amorphous, 150 °C annealed and 300 °C annealed samples is shown in Fig. 2d, with the samples clearly distinguishable and visible. These results indicate that compared with a GST-based display, double layer PCMs (Sb_2_Te_3_ and GeTe) can be employed for a better colour depth modulation, i.e., tunable multiple colours via sequential crystallization.

**Figure 3:**
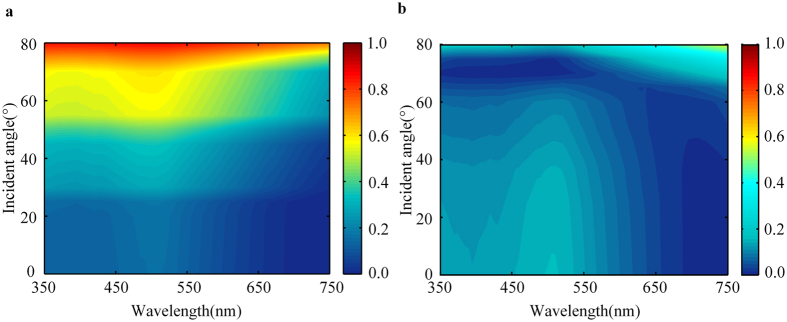
Reflectivity spectra. (**a**,**b**) Calculated reflectivity spectra for TE and TM polarization, respectively, for angles of incidence from 0° to 80° for the amorphous sample (the reflectivity value is described by the colour bars).

For the display device, it is very important that the optical properties are robust with respect to the angle of incidence. To understand the relationship between the colour appearance of the device and the incident angle, we systematically calculated the reflectivity spectra of the as-deposited amorphous sample at different angles of incidence from 0° to 80° for TE and TM polarization by using equation (4) and (5), respectively. The reflectivity spectra for TE polarization, as shown in Fig. 3a, indicates that the reflectivity peak wavelength remains unchanged for incident angles from 0° to 80°. In the case of TM polarization (Fig. 3b), the reflectivity feature is prominent for angles lower than 60°. It can be seen that the optical properties remain prominent for angles of incidence from 0° to 60° in both polarization. This is the reason why the colour of the device does not change when the devices are viewed from large angles of incidence. Because each layer of the device is much thinner than the wavelength of visible light, the phase accumulation propagating through the film is small compared to the reflection phase change on reflection^2^. As a result, the optical properties of the device show a low sensitivity to the angle of incidence.

### Laser-induced colour modulation

**Figure 4:**
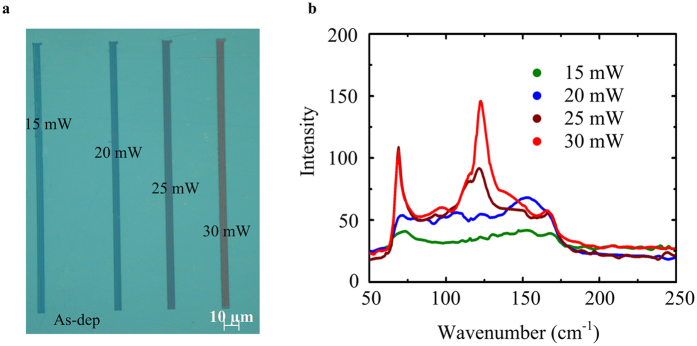
Colour modulation induced by laser illumination. (**a**) Microscope images of laser-induced colour modulation on the as-deposited amorphous sample. The laser power is 15 mW, 20 mW, 25 mW and 30 mW. (**b**) The Raman spectroscopy measurements of four lines correspond to the thermally crystalline samples shown in Fig. 2c.

Next, we demonstrate that the colour of the stack with double layer phase change films can be continuously modulated optically for the use in future displays. Laser illumination has been used in many ways, for example in optical media and in display and phase-change lithography^29^,^30^,^31^, hence, we employ this technique to demonstrate our optically enabled display technology. The final colour appearance is associated with the amount of crystallized PCMs in the illuminated area. In our experiment, the amount of crystallized PCMs is determined by the laser power. The as-deposited amorphous sample was fixed on a high-resolution X-Y stage driven by a stepping motor (details see Supplementary Fig. S1). The light from the wavelength of 661 nm that was emitted from the semiconductor laser (Cube-100C, Coherent Co., USA) was focused on the sample surface using an auto-focus system (SGSP-OBL-3, Sigma KOKI Co., Japan). To present the colour modulation, a continuous-wave laser with gradient power was used to irradiate the surface of the as-deposited amorphous sample. The laser power was 15 mW, 20 mW, 25 mW and 30 mW. The optical microscope image presented in Fig. 4a shows that the application of four different laser power magnitudes created four line patterns with a length of approximately 190 μm. Obviously, the colour becomes closer to that of the fully crystalline sample as the laser power increases. For a better visualization of the crystallization that take place during the laser illumination, Raman spectroscopy measurements were performed on the four lines. The results presented in Fig. 4b show an excellent correspondence to those of the three samples shown in Fig. 2c, proving that the colour change of the four lines is associated with the crystallization of double layer PCMs. Consequently, the colour is continuously modulated optically on a single device between the fully amorphous and the fully crystalline allowing for the creation of vivid colours without the need for colour filters or a spatially modulated colour scheme.

### Colour gamut

**Figure 5:**
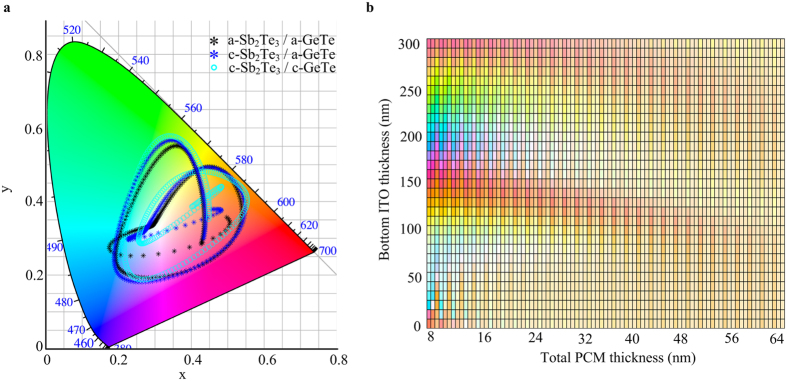
Colour gamut. (**a**) Obtainable nearly the entire colour gamut with 30 nm ITO/2 nm Sb_2_Te_3_/6 nm GeTe/t nm ITO/100 nm Au stacks, t is the only thickness that is varied. The bottom ITO thickness t is swept from 0 nm to 300 nm in steps of 1 nm. (**b**) The resulting collection of colour points of ITO/Sb_2_Te_3_/GeTe/ITO/Au stacks with increasing bottom ITO and PCMs.

As it was verified in Fig. 2b, optical simulations provided a reasonable match with the experimental results. Therefore, additional simulations were carried out to validate the feasibility of various colour expressions by combining with different thicknesses of the bottom ITO layer and selective crystallization of PCM layers. The colour performance of GST-based device has been reported previously^32^, here we demonstrate that nearly the entire colour gamut is theoretically available by exploiting double layer PCMs (Sb_2_Te_3_ and GeTe). Colour depth modulation capability has also been improved (tunable three colours) by adding another phase change film. We use the transfer matrix in equation (1) to study the similarities in colour generation and modulation in devices containing double layer PCMs with a variety of thicknesses of bottom ITO layer. The reflectance curve and its shift tendency with respect to the selective crystallization of PCM layers for 60 nm, 120 nm, 180 nm and 250 nm ITO are presented (see Supplementary Fig. S2). The optical reflectivity changes dramatically between different phases of double layer PCMs for every thickness ITO. It can be seen that the difference in thicknesses of bottom ITO layer resulted in shifting the position of maxima and minima of the reflectance spectra. The selective crystallization of Sb_2_Te_3_ layer leads to a blue-shift in the spectrum. When both Sb_2_Te_3_ and GeTe layers are crystallized, the reflectance curve is further shifted to shorter wavelengths. The reason of the different reflectivity spectra for different thicknesses ITO is the constructive and destructive interference between the Au layer and the ITO/GeTe/Sb_2_Te_3_ interface. To predict the final perceived colour by the human eye upon switching, we develop a model from the simulated stacks. By combining with the thickness and refractive index data of known materials, we calculate the reflectivity using the transfer matrix model and convert the results into colour coordinates, i.e., tristimulus values^33^. The colour generated by all possible combinations of Sb_2_Te_3_, GeTe and bottom ITO is calculated by using the standard illuminant D65 and the colour matching functions of the 1931 standard observer. Subsequently, we plotted the XY colour gamut on a chromaticity diagram as shown in Fig. 5a. In combination with the thickness of the bottom ITO layer, the phases of double layer PCMs can generate varieties of different colours. It is important that wide range of colour can be obtained by the stack based on double layer PCMs. Figure 5a shows the Red, Green, Blue values in the CIE space can be obtained for the 2 nm Sb_2_Te_3_ and 6 nm GeTe. The three representative values represents the three different thicknesses of the bottom ITO layer. As a result, nearly the entire colour gamut is available with the stack. Figure 5b shows the resulting collection of colour points. The thickness of Sb_2_Te_3_ varies from 2 nm to 30 nm and that of GeTe varies from 6 nm to 34 nm (The total PCMs thickness increases from 8 nm to 64 nm). Each PCM thickness point represents the calculated colour for the a-Sb_2_Te_3_/a-GeTe (left), c-Sb_2_Te_3_/a-GeTe (center) and c-Sb_2_Te_3_/c-GeTe (right) case next to each other in a amoamo/cryamo/crycry/amoamo/cryamo/crycry/etc. sequence. With the increasing thickness of Sb_2_Te_3_ and GeTe, the ability to generate colour degrades rapidly. The colour contrast between different phases of double layer PCMs also decreases with the increasing thickness of Sb_2_Te_3_ and GeTe, which is in agreement with the results shown in Fig. 2a.

## Discussion

A reflective-type display device based on phase change materials is attractive but the colour depth modulation is still not completely resolved. Double layer PCMs were suggested to obtain multiple colours by selective phase transition of each PCM layer instead of single PCM film. The colour could be continuously modulated optically for the use in future displays. Previous studies used two ultrathin GST films to achieve the multiple optical state with need for a thermal/diffusion barrier layer. One concern about the role of thermal barrier layer is its possible thermal insulation effect during the reset (amorphization) process, which will adversely interfere with the amorphization during actual device operation^10^. Our results suggested that Sb_2_Te_3_ and GeTe layers could crystallize sequentially without a barrier layer and also obtain a continuous colour modulation optically.

In summary, we have demonstrated that double layer PCMs (Sb_2_Te_3_ and GeTe) can be employed to obtain a better colour modulation capability than that of a GST-based device. The sequential crystallization can be achieved stably without a barrier layer due to the Tc difference between Sb_2_Te_3_ and GeTe instead of accurate thickness control, resulting in high contrast multiple colours. The optical contrast of Sb_2_Te_3_ and GeTe integrated in this structure is optimized. Furthermore, the optical properties of the stack are robust with respect to the angle of incidence, which is important for display applications and colour consistency on flexible substrates. The non-binary colour rendering on a single device by optical excitation is demonstrated for the first time. In combination with the thickness of the bottom ITO layer and selective phase transition of PCM layers, nearly the entire colour gamut is feasible with this technology. These results pave the way for a new generation of display technologies. For future displays, the use of PCM with lower crystallization time and power may prove to be beneficial for achieving reduced power consumption.

## Methods

### Film deposition

Films were sputtered directly on an Si substrate covered with a 1 μm layer of thermally grown SiO_2_. 10 nm Ti and 100 nm Au layers were deposited using electron beam evaporation from an Oxford evaporator. A Ti film was used to improve the adhesion between Au and substrate. The samples were then moved to a magnetron sputtering system (JZCK-640, JUZHI Co., China) for ITO, GeTe and Sb_2_Te_3_ deposition. ITO was sputtered at 50 W DC power and 0.4 Pa Argon pressure at a rate of 2.7 nm min^−1^. For GeTe and Sb_2_Te_3_ deposition, the sputter was set to DC 30 W and RF 70 W, 0.6 Pa Argon pressure at a rate of 13.3 nm and 7.2 nm min^−1^, respectively.

### Reflectance measurements

Reflectance values of different samples were measured using a Lambda750s spectrometer (Perkin Elmer, USA) at an incident angle of 0° and in the wavelength range between 350 and 750 nm.

### Photographs

Photographs were taken with a Nikon D3200 camera with a AF-S 50 mm f/1.8 G lens.

### Microscopy

Microscope images were taken with a Carl Zeiss Axiovert 200 MAT light microscope.

### Raman spectroscopy

The Raman measurements were performed with a green laser at 532 nm. The laser intensity was reduced to 0.1 mW μm^−2^ for the sample to avoid crystallization.

## Additional Information

**How to cite this article**: Ji, H.-K. *et al*. Non-binary colour modulation for display device based on phase change materials. *Sci. Rep.* **6**, 39206; doi: 10.1038/srep39206 (2016).

**Publisher's note:** Springer Nature remains neutral with regard to jurisdictional claims in published maps and institutional affiliations.

## Supplementary Material

Supplementary Information
