# Supplementary material for: Non-binary Colour Modulation for Display Device Based on Phase Change Materials
Source: Sci Rep. 2016 Dec 19;6:39206. doi: 10.1038/srep39206 (PMC5171701; doi:10.1038/srep39206)
Supplement: Supplementary Information [file srep39206-s1.pdf]

Supplementary Information

Non-binary Colour Modulation for Display Device Based on Phase Change Materials

Hong-Kai Ji[1†], Hao Tong[1†], Hang Qian[1], Ya-Juan Hui[2], Nian Liu[1], Peng Yan[2], Xiang-Shui Miao[1,2*]

1.School of Optical and Electronic Information, Huazhong University of Science and Technology, Wuhan 430074, China.

2.Wuhan National Laboratory for Optoelectronics, Huazhong University of Science and Technology, Wuhan 430074, China.

*Corresponding author. Email: miaoxs@hust.edu.cn

†These authors contributed equally to this work.

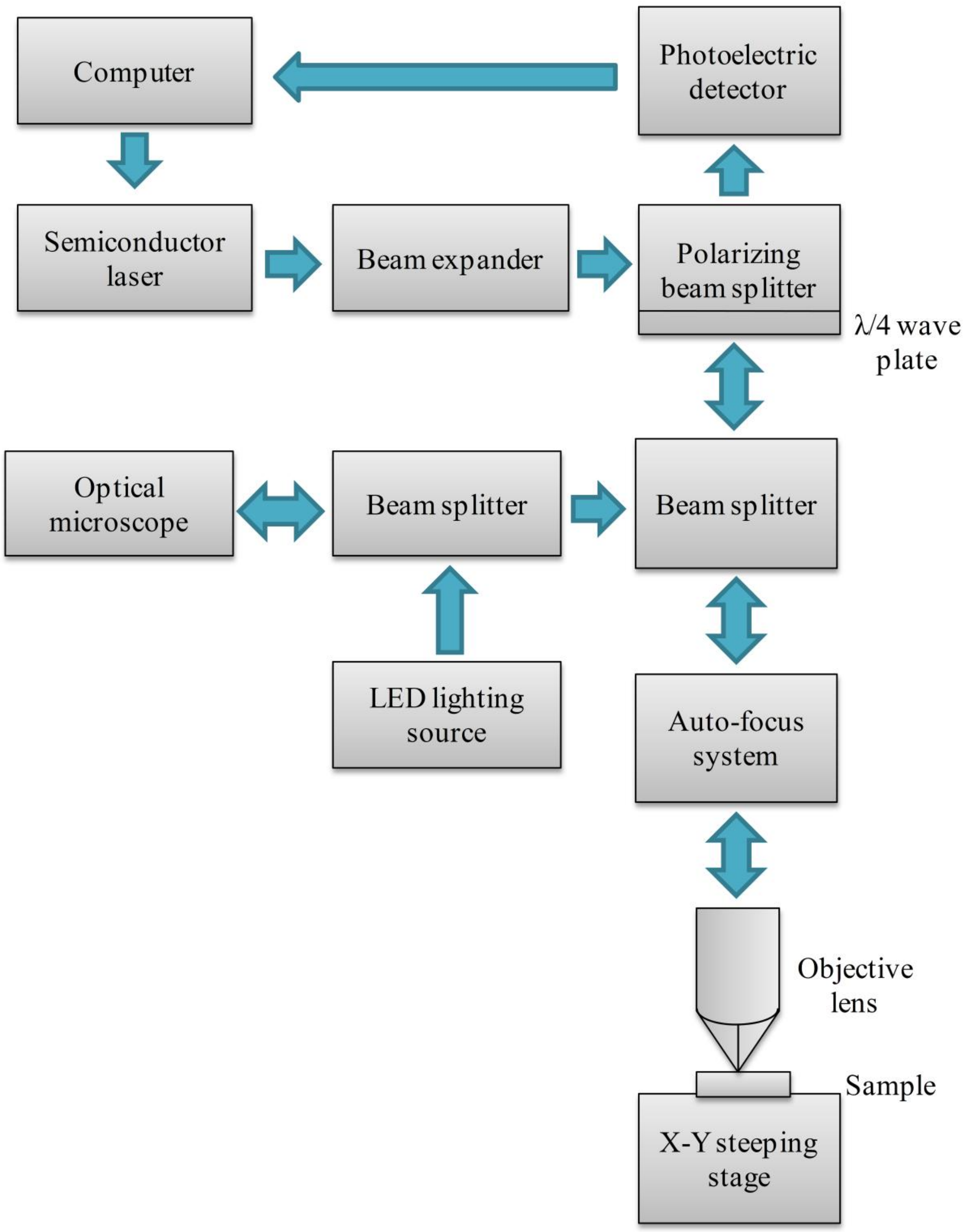

Supplementary Figure S1. The schematic of exposure system. The wavelength of semiconductor laser is 661 nm and the numerical aperture of the objective lens is 0.4. The high-resolution X-Y stage is driven by a stepping motor. With the need of beam expander, beam splitter and auto-focus system, the samples are focused accurately. Line patterns and dot patterns can be created using continuous wave and pulse laser, respectively. Laser power, pulse width and movement speed of the X-Y stage are variable. The laser spot diameter is 1 μm and the movement speed of the X-Y stage is set to 62.5 μm/s. In the laser-induced colour modulation process, the sample is illuminated from top

to bottom for 190 μm in the Y direction first to generate a line pattern with the width of 1 μm approximately. To avoid illuminating the crystalline line pattern again, the sample moves 1.25 μm in the X direction. Then, the sample is illuminated from bottom to top for 190 μm in the Y direction for another crystalline line pattern. Repeat this process for a wide line. Each wide line of different laser power consists of 4 crystalline lines for a visible colour.

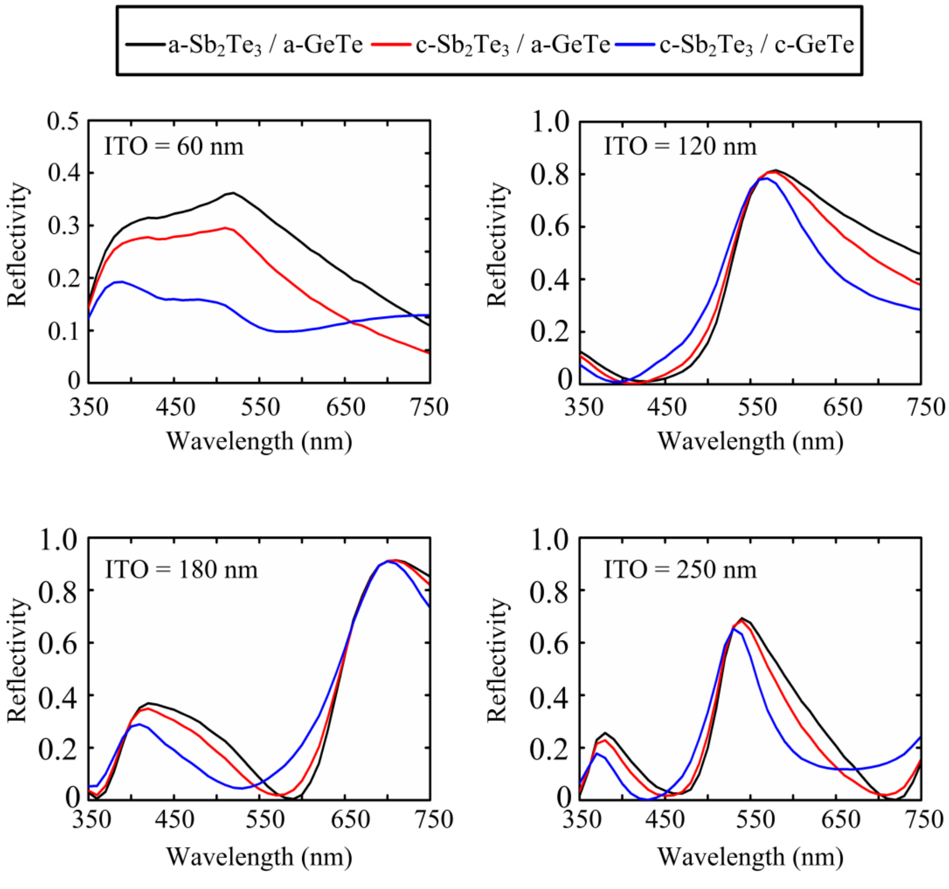

Supplementary Figure S2. The simulated reflectivity of different phases for various thicknesses of the bottom ITO layer in stack (30 nm ITO/2 nm Sb_2Te_3/6 nm GeTe/t nm ITO/100 nm Au, t is the only thickness that is varied). The reflectivity spectra of 60 nm, 120 nm, 180 nm and 250 nm ITO are shown in Figure S2. The optical reflectivity changes dramatically between different phases of double layer phase change materials for every thickness ITO.
